# SARS-CoV-2 Related Myocarditis: What We Know So Far

**DOI:** 10.3390/jcm12144700

**Published:** 2023-07-15

**Authors:** Raffaella Mistrulli, Armando Ferrera, Melwyn Luis Muthukkattil, Massimo Volpe, Emanuele Barbato, Allegra Battistoni

**Affiliations:** 1 Clinical and Molecular Medicine Department, Sapienza University of Rome, 00185 Roma, Italy; armando.ferrera95@gmail.com (A.F.); massimo.volpe@uniroma1.it (M.V.); emanuele.barbato@uniroma1.it (E.B.); allegra.battistoni@uniroma1.it (A.B.); 2 IRCCS San Raffaele, 00163 Roma, Italy

**Keywords:** COVID-19 related myocarditis, cardiovascular sequelae of COVID-19, post-acute sequelae of SARS-CoV-2, COVID-19 and myocarditis, COVID-19–associated acute myocarditis, COVID-19, acute myocarditis, SARS-CoV-2

## Abstract

A minority of patients with severe acute respiratory syndrome coronavirus 2 (COVID-19) develop cardiovascular complications, such as acute cardiac lesions with elevated troponins, de novo systolic heart failure, pericardial effusion and, rarely, acute myocarditis. The prevalence of COVID-19-related myocarditis ranges from 10 to 105 cases per 100,000 COVID-19-infected individuals, with a male predominance (58%) and a median age of 50 years. The etiopathogenetic mechanism is currently unclear, but may involve direct virus-mediated damage or an exaggerated immune response to the virus. Mortality is high, as fulminant myocarditis (FM) develops very often in the form of cardiogenic shock and ventricular arrhythmias. Hence, medical therapy with ACE inhibitors and beta-blockers may not always be sufficient, in which case inotropic and immunosuppressive drugs, most commonly corticosteroids, may be necessary. In this review we analyze the current data on COVID-19 myocarditis, management strategies and therapy, with a brief description of COVID-19 vaccine-associated myocarditis to help clinicians dealing with this peculiar form of myocarditis.

## 1. Introduction

CoronaVIrus Disease 19 (COVID-19) has emerged as a major cause of morbidity and mortality worldwide [1,2]. The range of its clinical presentations consists of cough, fever, fatigue, dyspnea, anosmia, ageusia, myalgia and pharyngodynia, but it may also dramatically evolve into acute respiratory distress and multiorgan failure [3,4]. Although COVID-19 has been primarily considered a respiratory disease [5], it is now recognized as a complex pathological condition that may involve several systems, including the cardiovascular system, which represents its second major target [6]. Among cardiac manifestations of COVID-19, acute myocarditis (AM) appears to be one of the most clinically significant, as it triggers a sudden deterioration of patients’ clinical status and may lead to death [7]. Indeed, in a large multinational registry of COVID-19 patients, an association was observed between myocarditis diagnosis and increased mortality [7]. Despite the clinical relevance of myocarditis, its incidence is still debated due to a high number of mildly symptomatic cases that might go undetected. Moreover, the pathogenesis is not completely understood.

Comprehensive management of COVID-19 associated myocarditis, from diagnostic tools to specific treatments, is crucial to avoid repercussions on patients’ health.

Therefore, the following narrative review aims to summarize the current literature on the relationship between COVID-19 and AM with regards to its epidemiology, pathogenetic mechanisms, diagnostic and prognostic features, and possible therapeutic approaches. The review also discusses SARS-CoV-2 vaccination-related AM. For our literature search, we used the following terms: “COVID-19 related myocarditis” or “Cardiovascular Sequelae of COVID-19” or “Post-Acute Sequelae of Severe Acute Respiratory Syndrome Coronavirus 2 (SARS-CoV-2)” or “COVID-19 and myocarditis” or “COVID-19–Associated Acute Myocarditis” and “COVID-19/myocarditis and SARS-CoV-2”. We included articles published from 2020 to 1 March 2023, in English, on both PubMed and MEDLINE. We reviewed original articles and meta-analyses and selected the most recent papers and those with the largest sample size. Reviews, consensus papers, and guidelines were included when deemed relevant and related to the topic.

## 2. Myocardial Injury during COVID-19 and COVID-19-Related Myocarditis: Epidemiological Aspects

According to the Fourth Universal Definition of Myocardial Infarction, acute myocardial injury (MI) is defined by a rise and/or fall of cardiac troponin (cTn) values with at least one value above the 99th percentile upper reference limit [8]. Conversely, AM is a disease characterized by a specific clinical presentation, with cardiac symptoms such as acute/new-onset chest pain or signs of left and/or right heart failure and/or unexplained arrhythmias or aborted sudden death, in addition to elevated cTn values and abnormal electrocardiographic/echocardiographic/cardiac magnetic resonance (CMR)/histopathologic findings, in the absence of coronary artery, valvular, or congenital heart disease [9]. MI is more common in COVID-19 patients than in patients with other severe diseases caused by different respiratory viruses [10]. It occurs in about 20% of hospitalized COVID-19 patients and in about 40–50% of critically ill COVID-19 patients [11,12]. A recent meta-analysis by Santoso et al., shows that MI is a frequent occurrence in COVID-19 and a poor prognostic factor, as it is associated with a higher risk of malignant arrhythmias, need for intensive care, shock, and death [13]. It is, therefore, crucial to recognize and diagnose it during COVID-19 in order to identify “high-risk” patients and limit its consequences on patients’ health [14]. According to the most recent data, the prevalence of myocarditis has been estimated to be about 2/100,000 patients [15]. Similar data found by Golpour et al. showed that the worldwide prevalence of AM ranges from 10 to 105 cases per 100,000 subjects depending on geographic differences and locally available diagnostics procedures [16]. The incidence and prevalence of COVID-19-related myocarditis is rarely investigated because of different—and often unclear—definitions and due to the lack of systematic data collection during the recent pandemic. According to a population-based analysis conducted by Singer et al. [17] involving young adults from 48 United States healthcare organizations, the incidence of myocarditis during SARS-CoV-2 infection amounted roughly to 45 per 100,000 patients. In a recent multinational, retrospective study by Ammirati et al. involving 23 cardiology centers, the estimated prevalence of COVID-19-related myocarditis was about two cases per 1000. Regardless of the context, SARS-CoV-2-related AM is more common in men [14,16,18,19].

## 3. Diagnostic Evaluation in COVID-19-Related Myocarditis

In most patients affected by COVID-19, AM occurs during the acute infection. Clinical presentation, electrocardiography, echocardiography, and laboratory tests, as well as cardiac magnetic resonance (CMR), are the cornerstone tools for diagnosis [20]. In patients affected by SARS-CoV-2 infection with a low clinical suspicion for cardiac involvement, no further cardiac testing is needed. Chest pain or discomfort, dyspnea, palpitations, and syncope are suggestive of cardiac involvement during COVID-19 [21]. In patients with symptoms indicative of cardiac involvement, a 12-lead electrocardiogram (ECG), an echocardiogram, and blood samples for cardiac troponin are indicated [21]. Electrocardiographic abnormalities in myocarditis are not pathognomonic, but sinus tachycardia, ectopic ventricular beats, ST segment elevation without reciprocal ST segment depression, diffuse T-wave inversion, QT interval prolongation, QRS prolongation, low voltages, atrioventricular block, bundle branch block and ventricular tachycardia may be present [22,23]. The most common electrocardiographic abnormalities are ST segment alterations and sinus tachycardia, which are both reported in 38% patients with COVID-19 related myocarditis. T wave abnormalities are described in about one fifth of patients with COVID-19 related myocarditis, whilst low QRS voltage or low QRS progression are reported only in 17% of patients. Ectopic rhythm origin and ventricular tachycardia are increasingly rare and reported respectively in 12% and 7% of patients affected by COVID-19 related myocarditis. In about 5% of patients, no pathological electrocardiographic features may be found [20]. 

Echocardiography is the first line imaging test in COVID-19 related myocarditis, with common echocardiographic alterations being regional hypokinesia (reported in 36% of patients), systolic dysfunction (reported in 83% of patients), left ventricular diastolic dysfunction, and impaired left ventricular myocardial deformation (abnormal ventricular strain) [24,25]. Pericardial effusion has been described in 29% of COVID-19-related myocarditis patients, while mitral or tricuspid regurgitation is reported in only 6% of patients [20]. Inflammatory markers, such as C-reactive protein (CRP), that is found to be increased in about 95% of patients with AM, as well as procalcitonin, ferritin, and interleukin-6, are usually elevated, together with the white blood cell count. Cardiac biomarkers, such as D-dimer, N-terminal brain natriuretic peptide (NT-pro-BNP) or brain natriuretic peptide (BNP), and cardiac troponins (cTn), are elevated in almost 95% of patients. Their rise is indicative of a hyperinflammatory state and is a surrogate of myocardial injury, suggesting a possible diagnosis of AM [20,26]. In such cases, additional tests can be used to rule out AM and/or to diagnose other pathological conditions, such as invasive coronary angiography (ICA) or computed tomography coronary angiography (CTCA) to exclude ischemic heart disease and computed tomography pulmonary angiography (CTPA) to exclude pulmonary embolism. If clinically stable COVID-19-related myocarditis is suspected, cardiac magnetic resonance (CMR) imaging should be considered in order to confirm the diagnosis of myocarditis or other myocardial (and pericardial) diseases [27]. The Lake Louise criteria for myocarditis have been fulfilled in about two-thirds of patients affected by COVID-19-related myocarditis [20]. Non-ischemic myocardial injury characterized by sub-epicardial or transmural late gadolinium enhancement (LGE) and myocardial edema are the most common CMR features observed in COVID-19-related myocarditis [28,29]. Endomyocardial biopsy (EMB) is considered the gold-standard for myocarditis diagnosis, but it is rarely used in clinical practice as it is an invasive procedure [30]. Moreover, it is associated with a high false-negative rate and a rate of complications of about 6% [31,32]. Hence, in cases of suspected COVID-19-related myocarditis, EBM should be considered only in hemodynamically unstable patients or those with rapid clinical deterioration after exclusion of coronary artery disease [21] (Table 1).

## 4. Pathophysiology of COVID-19 Related Myocarditis

**Figure 1:**
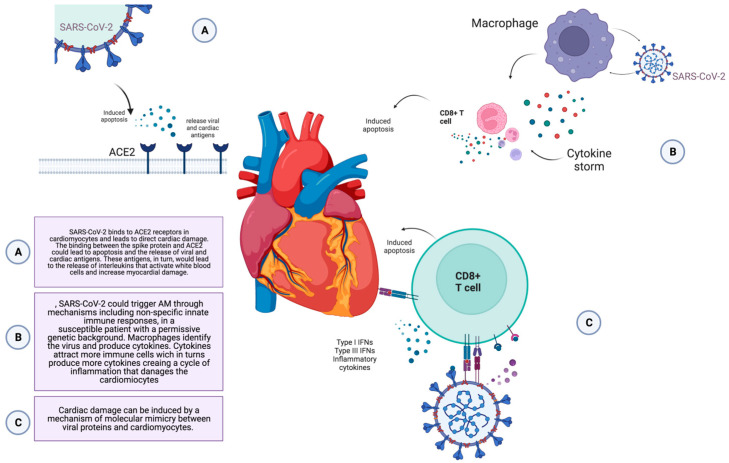
Potential pathogenetic mechanisms involved in COVID-19 related myocarditis. ACE 2—the angiotensin-converting enzyme 2 receptors. Created with Biorender.com, accessed on 23 April 2023.

The pathogenesis of COVID-19-related myocarditis is currently unknown. Direct cardiac injury by SARS-CoV-2 has been hypothesized as studies have shown that the virus can bind its spike protein to angiotensin-converting enzyme 2 (ACE2) receptors on ciliated columnar epithelial cells of the respiratory tract, type II pneumocytes, and cardiomyocytes. Therefore, SARS-CoV-2 may directly infect the heart, especially in the setting of pre-existing heart failure, where ACE2 receptors are upregulated, despite the presence of viral receptors not being accurate predictors of tropism [33]. The binding between the spike protein and ACE2 leads to apoptosis and to the release of viral and cardiac antigens. These antigens, in turn, cause the release of interleukins (IL-1, IL-6, IL-12, TNF-alpha) that might activate T lymphocytes and increase myocardial damage through cell-mediated cytotoxicity, leading to a vicious cycle of immune activation and myocardial injury [34]. However, currently available data suggest other hypotheses since myocardial biopsies in patients with COVID-19-related myocarditis have rarely documented viral presence in cardiac cells [14,35]. In most cases, an infiltration with interstitial macrophages and more rarely lymphocytes have been found [36,37]. Therefore, it seems reasonable that COVID-19 could trigger AM through a non-specific innate and/or adaptive immune response in genetically susceptible patients [38]. A recent analysis showed an increased risk of arrhythmias and myocarditis up to 12 months after infection [39,40]. Therefore, a third hypothesis of a molecular mimicry between viral proteins and cardiomyocytes has been developed. This latter might require a longer time between exposure to the virus and the development of cardiac damage. In conclusion, given the paucity of published data and the heterogeneous results, the mechanisms through which SARS-CoV-2 can induce myocardial damage and inflammation are yet to be outlined (Figure 1)

## 5. Prognosis of COVID-19 Related Myocarditis

Previous reports, such as the Lombardy registry on non-COVID-19-related myocarditis, found that fulminant myocarditis (FM) accounted for 8.3% of cases [14,41], whereas in a multicentre study conducted by Ammirati et al., a high percentage (38.9%) of patients with COVID-19-associated myocarditis had FM (cardiogenic shock and arrhythmias) [14]. A recent registry report confirmed gigantocellular myocarditis as having the highest mortality or need for HTx (81% at 3-year follow-up), followed by eosinophilic myocarditis in the context of systemic inflammatory diseases or drug allergies such as DRESS (drug rash with eosinophilia and systemic symptoms). Lymphocytic FM also proved to be a high-risk condition, with a death or HTx rate of 19.5% at 60 days and 40% at 3 years [42]. Its cause is often viral, as shown by a systematic review that showed that AM from influenza viruses was associated with an overall mortality rate of 14.7% [43]. Patients with COVID-19-associated myocarditis had a higher mortality rate; in fact, 18.5% of patients with FM and cardiogenic shock required temporary mechanical circulatory support (t-MCS). According to the available evidence, left ventricular EF is frequently low on the first echocardiogram, and the evolution to a fulminant form is not related to the presence of pneumonia [14]. This is of particular interest, since the severity of myocarditis triggered by SARS-CoV-2 seems also to be independent from the occurrence of viral pneumonia, despite patients with associated pneumonia usually having a worse prognosis [14]. In a review of 38 published cases of suspected myocarditis with COVID-19, the mortality rate was very high (13.8%), although there may be a publication bias that led to an overestimation of mortality [26]. However, the high mortality rate may also be explained by milder forms of AM being underestimated, unrecognized, and/or not attributed to COVID-19. In this regard, in two studies that evaluated cardiac involvement in athletes with COVID-19, myocarditis was often asymptomatic or paucisymptomatic and in a mild form (the lowest EF found at CMR was 41%). After several months of follow-up, none of the patients had any adverse cardiac events [18,44]. Further studies providing more information on both in-hospital and long-term mortality and cardiovascular complications are required. 

## 6. Treatment of COVID-19 Related Myocarditis

No randomized controlled trials have tested specific therapies for AM associated with COVID-19, with the available data being based on observational studies. In general, the management is the same as that of heart failure, including pharmacological therapy, fluid management, and advanced therapies in cases of unresponsive or cardiogenic shock [45]. The use of angiotensin-converting enzyme inhibitors (ACE-I) or angiotensin receptor blockers (ARB) together with mineralocorticoid receptor antagonists (MRA) is considered to be a part of the management of patients affected by COVID-19-related myocarditis [46]. The same applies to beta-blockers (BBs), which are indicated for their anti-arrhythmic effect, especially in the presence of reduced EF. From the data of the Lombardy registry, BBs were among the most prescribed drugs in patients with AM regardless of etiology [41], and in the study conducted by Ammirati et al., BBs were also used in 55.5% of patients [14].

This analysis also showed a high number of FM requiring inotropes and/or vasopressors (38.9%). Immunosuppressive therapies were used in 59.2% of cases, with a high prevalence of intravenous corticosteroid use, independently from the presence or absence of pneumonia (43.4% and 48.3%, respectively (*p* = 0.79)). In some cases, a combination of corticosteroids and intravenous immunoglobulins (IVIG) or tocilizumab was used, while IVIG alone was administered in only two patients [14]. The clinical benefit of corticosteroids in hospitalized patients with COVID-19 has now been shown in some trials, but in the specific context of AM data are lacking [47,48,49]. Small case series have shown a favorable prognosis associated with the use of intravenous corticosteroids in adults with COVID-19 multisystem inflammatory syndrome (MIS) and myocarditis [39,48]. Several immunosuppressive therapies, such as interleukin-1 (anakinra and canakinumab) and interleukin-6 (tocilizumab and sarilumab) antagonists, have demonstrated promising results in selected patients hospitalized for COVID-19 [50,51,52,53]. However, none of these drugs have been investigated specifically for COVID-19-related myocarditis. In summary, corticosteroids may be a choice in hospitalized patients with myocarditis and COVID-19, regardless of pneumonia and hypoxemia, but should be avoided in less severe forms [45].

## 7. Update of Myocarditis after SARS-CoV-2 Vaccination

**Figure 2:**
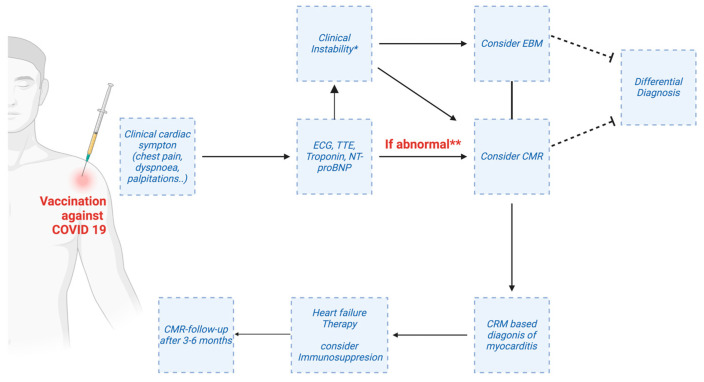
Potential workflow for the use of advanced cardiac magnetic resonance (CMR) in patients post-vaccination and suspected myo-/pericarditis. ECG—electrocardiogram; EBM—endomyocardial biopsy; NT-proBNP—N-terminal pro-B-type natriuretic peptide; TTE—transthoracic echocardiography. * e.g., Severe arrhythmias, worsening heart failure. ** always rule out other causes of troponin elevation (e.g., myocardial infarction, pulmonary embolism.). Created with Biorender.com.

In the past, vaccination-associated myocarditis was a very rare adverse event that occurred following the administration of the smallpox attenuated virus vaccine and, less commonly, after other vaccines, such as those for diphtheria, tetanus, polio, influenza and hepatitis B [54]. With the implementation of the COVID-19 vaccination campaign, several countries introduced reporting systems for vaccine-adverse events, such as VAERS that monitored outcomes in approximately 200 million individuals in the United States. Using data from this large database, a small but stable rate of patients presented with post-vaccination myocarditis and/or pericarditis. By June 2021, a total of 1226 cases of suspected vaccine-associated pericarditis and myocarditis had been recorded in VAERS, with 40% of the events having occurred in individuals aged ≤30 years [55]. Of these, 323 reports were confirmed by CDC (Centers for Disease Control and Prevention) [56]. The estimated rate of myocarditis/pericarditis in subjects aged <30 years after receiving the second dose of the mRNA vaccine was approximately 40 cases per million among men and 4.2 cases per million among women. When considering the age group ≥ 30 years, these rates were significantly reduced to 2.4 and 1.0 per million in men and women, respectively [57]. The most recent US data showed that incidence peaks in young males aged 15–17 years, with 105.9 cases per million doses administered, and identified the second dose as being associated with the highest risk [54]. The true incidence of vaccination-related myocarditis is unknown, as available reports only refer to symptomatic patients. To estimate the true incidence, a systematic evaluation by means of instrumental and laboratory examinations should be performed in a larger population of individuals who have received the vaccine [57]. Reports about myocarditis after SARS-CoV-2 vaccination are heterogeneous due to different inclusion criteria in studies, different characteristics (age, sex, etc.) of the subjects, different criteria to diagnose post-vaccination myocarditis, different types and doses of vaccines, and different follow-up periods after vaccination. Simone et al. found post-vaccination myocarditis to be a very rare event (0.58 per 100,000 individuals vaccinated with a second dose) in a multiethnic population (31.2% “White”, 6.7% “Black”, 37.8% “Hispanic”, 14.3% “Asian”) after a follow-up of 10 days from the second dose [58]. From a review of 90 cases published in the literature [59], the median age was 25 years (interquartile range 17–27)—comparable to that observed in VAERS [60]—and with a marked male prevalence (93%). In most subjects, myocarditis occurred after the second dose, with a mean time of 3 days between the last dose and the onset of symptoms. These results suggested an immune-mediated reaction to vaccine administration [54]. Indeed, the proposed mechanisms by which SARS-CoV-2 vaccines could induce myocarditis might involve the activation of both innate and adaptive immune responses against the SARS-CoV-2 spike glycoprotein, but also the recognition of the mRNA itself as an antigen by the immune system [55]. Since, like other viral myocarditis, post-vaccine myocarditis occurs predominantly in young men, sex hormones may play a role in genetically susceptible individuals. With regards to the clinical presentation, the most frequent symptom was chest pain (in 96% of cases) generally preceded by fever (in 85%). The ECG was altered (mild, diffuse ST-segment changes, PQ-segment depressions or non-specific ST-segment changes) in 77% of patients, while the echocardiogram showed only a slight reduction in left ventricular EF (mean EF of 53%), with pericardial effusion in less than one third of patients [60]. CMR was often altered, with a pattern suggestive of myocarditis (according to the updated Lake Louise Criteria). EBM was rarely performed, most often with negative or non-specific histological findings [61,62,63,64,65]. Concerning therapy, non-steroidal anti-inflammatory drugs (NSAIDs) were most commonly used, followed by corticosteroids and, in a smaller percentage, by IVIG. Colchicine and anakinra were rarely used. In most cases, immunosuppressive agents were used in combination [61]. The prognosis is generally favorable. In a case series of 139 adolescents (all <21 years of age) with suspected myocarditis within 30 days of COVID-19 vaccination, none died or required t-MCS. No patients had cardiac events at 6 months of follow-up [66]. An analysis of the Israeli “Clalit Health Services” registry compared a cohort of over 800,000 BNT162b2 (Pfizer) mRNA-vaccinated individuals aged 16 years and older with an unvaccinated cohort of patients with SARS-CoV-2 infection documented by polymerase viral chain reaction (PCR). The cohorts were followed up for 42 days, starting on the day of administration of the first dose of COVID-19 vaccine. The cohort of unvaccinated SARS-CoV-2 infected patients experienced a significantly higher risk of myocarditis than the cohort of BNT162b2 mRNA-vaccinated subjects in the order of 11 cases vs. 2.7 cases per 100,000 individuals, respectively. In addition, the vaccinated cohort developed a mild and rapidly resolving myocarditis, whereas the unvaccinated cohort had an increased risk of other serious cardiac complications, such as arrhythmias and myocardial infarction. Despite its limitations, the results showed a risk-benefit profile strongly in favor of vaccination against COVID-19 [67] (Figure 2).

## 8. Conclusions

Although AM is not a frequent complication of COVID-19 infection, it is associated with a significant increase in morbidity and mortality. Its incidence is likely to be underestimated as there is no uniformity in diagnostic criteria. A central role in diagnosis is played by CMR and, where necessary, also by EMB. Currently, the management of SARS-CoV-2 myocarditis follows standard guidelines for heart failure, and immunosuppressive drugs have often been used with encouraging results. The use of corticosteroids, at least in severe forms, is still debated. Further studies are needed to fully understand the pathophysiological mechanisms and thus choose the most appropriate therapy. Although we have no long-term data, COVID-19 vaccine-associated myocarditis, on the other hand, is a rare, self-limiting event with a favorable prognosis. This confirms the benefits of COVID-19 vaccination over the risks associated with infection.

## Figures and Tables

**Table 1:** Comparison of patients’ features/ECG/echo findings at admission in patients affected by myocarditis according to a large recent registry and data available in patients affected by COVID-19-related myocarditis. ECG,—Electrocardiogram; LVEF—left ventricular ejection fraction.

	Acute Myocarditis (41)	SARS-CoV-2 Related Myocarditis (20)
Age at admission, median (years)	34	44
Male sex (%)	81%	73%
ECG alterations at admission (% of patients)	85%	95%
Echocardiography alterations at admission LVEF (mean) Presence of pericardial effusion (% of patients)	50% 25.7%	28% 6%
Abnormal troponin findings (% of patients)	99.3%	95%
